# A fast thermal-curing nanoimprint resist based on cationic polymerizable epoxysiloxane

**DOI:** 10.1186/1556-276X-7-380

**Published:** 2012-07-09

**Authors:** Jizong Zhang, Xin Hu, Jian Zhang, Yushang Cui, Changsheng Yuan, Haixiong Ge, Yanfeng Chen, Wei Wu, Qiangfei Xia

**Affiliations:** 1 Department of Materials Science and Engineering, College of Engineering and Applied Sciences, National Laboratory of Solid State Microstructures, Nanjing University, Nanjing, 210093, People's Republic of China; 2 Hewlett-Packard Laboratories, 1501 Page Mill Road, Palo Alto, CA, 94304, USA; 3 Department of Electrical and Computer Engineering, University of Massachusetts, Amherst, MA, 01003, USA

**Keywords:** Nanoimprint, Epoxysiloxane, Transfer layer, UV-assisted thermal curing, Cationic polymerization

## Abstract

We synthesized a series of epoxysiloxane oligomers with controllable viscosity and polarity and developed upon them a thermal-curable nanoimprint resist that was cross-linked in air at 110°C within 30 s if preexposed to UV light. The oligomers were designed and synthesized *via* hydrosilylation of 4-vinyl-cyclohexane-1,2-epoxide with poly(methylhydrosiloxane) with tunable viscosity, polarity, and cross-linking density. The resist exhibits excellent chemical and physical properties such as insensitivity toward oxygen, strong mechanical strength, and high etching resistance. Using this resist, nanoscale patterns of different geometries with feature sizes as small as 30 nm were fabricated *via* a nanoimprint process based on UV-assisted thermal curing. The curing time for the resist was on the order of 10 s at a moderate temperature with the help of UV light preexposure. This fast thermal curing speed was attributed to the large number of active cations generated upon UV exposure that facilitated the thermal polymerization process.

## Background

Resist is one of the key components for nanoimprint lithography (NIL) in addition to the imprint mold, the imprint machine, and imprint processes [1,2]. Depending on the process, nanoimprint resists can be classified into two basic categories: thermoplastic polymers [3] or thermal curable materials [4,5] for thermal-NIL, and UV-curable monomers or oligomers for UV-NIL [6-9]. The most commonly used NIL resists in earlier years were thermal plastic polymers, which required high pressure and high temperature so that the mold can be pressed into the molten resist film. Curing of thermal curable resists, although it requires lower pressure, is a time-consuming process due to the low speed of thermal-initiated polymerizations. Recently, UV-NIL has been developed for device fabrication, and it allows for imprinting at a low pressure and room temperature [6,10,11]. The process involves pressing a transparent mold into a low viscous photo-curable liquid thin film on a substrate and then solidifying the liquid materials *via* a UV light irradiation. The liquid resist can automatically fill the recess portions of the features on the mold due to capillary effect. An ideal UV resist usually has low viscosity, low surface tension, good adhesion to the substrate, fast cross-linking speed, high mechanical strength, and high etching resistance after cross-linking.

The fundamental ingredients of a UV resist include a UV-curable matrix, a photo-initiator, and other additives such as plasticizer, curing accelerator, photo-sensitizer, flow and leveling agent, fluorinated surfactant, and so on. Acrylate-based resins have been widely used in various UV-curable material systems as the backbone because of their great reactivity with a wide choice of acrylated monomers and oligomers [12,13]. However, a significant drawback of acrylated materials is that free radical polymerization is strongly inhibited by oxygen scavenging of the free radicals. This prevents UV-curing imprint process from being operated in ambient air environment, increasing the cost of equipment. To address this issue, materials such as vinyl ethers [14] and epoxides [15] based on cationic polymerization were used for UV-curable nanoimprint resists, which effectively cross-link upon UV exposure in the presence of air. However, vinyl ether resins are costly because of a narrow choice of commercially available monomers and oligomers. The cationic polymerization rate of epoxy groups is low, [16] and the conversion rate is less than 10% when the temperature is lower than 50°C [17,18]. Recently, new epoxy-based materials were developed for imprint resists [19,20]; the mechanism of the polymerization is not fully revealed. Furthermore, these resists can only be cross-linked by UV light, so a transparent mold or substrate is required. On the other hand, epoxy-based negative photoresist, such as SU-8, was used for combined UV and thermal NIL, but it required very high imprint pressure (as high as 30 bar) to press the mold into the molten resist [21-23].

Herein, we report on a new liquid NIL resist based on epoxysiloxane oligomers that can be thermally cured within a short period of time in air at a moderate temperature using a UV-assisted thermal curing process. We designed and synthesized these oligomers *via* hydrosilylation of 4-vinyl-cyclohexane-1,2-epoxide with Si-H group-functionalized siloxane precursor. The viscosity, cross-linking density, and polarity of epoxysiloxane were controlled on demand by employing siloxane precursors with varied molecular weights and Si-H functionality. We also developed a fast UV-assisted thermal imprint process that requires low pressure, moderate temperature without the demand for optically transparent mold and vacuum equipment. We fabricated nanostructures of different geometries and sizes with the resist and further demonstrated high aspect ratio pattern transfer by reactive ion etching (RIE).

## Methods

### Materials

Polymethylhydrosiloxane (PMHS) precursors with different Si-H functionality, (15% to 18% methylhydrosiloxane)-dimethylsiloxane copolymer, (50% to 55% methylhydrosiloxane)-dimethylsiloxane copolymer and polymethylhydrosiloxane terminated with trimethylsilyl, and p-(octyloxyphenyl)phenyliodonium hexafluoroantimonate (95%, photo-acid generator) were purchased from Gelest, Inc. (Morrisville, PA, USA). A 4-vinyl-1-cyclohexene 1,2-epoxide(VCHE) mixture of isomers, propylene glycol monomethyl ether acetate (PGMEA), and chlorotris(triphenylphosphine) rhodium(I) (Rh catalyst) were purchased from Sigma-Aldrich Corporation (St. Louis, MO, USA). Lift-off layer (LOL) 2000 was purchased from MicroChem Corp (Newton, MA, USA). All materials were used as purchased.

### Synthesis and characterization

**Figure 1:**
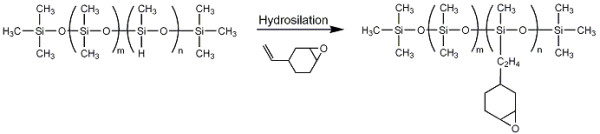
Synthesis of multifunctional epoxysiloxane through hydrosilylation reaction.

The hydrosilylation of olefinic functionalized compounds is one of the most reliable ways to introduce a desired functionality as a side- or an end-group on Si-H containing siloxane polymers [24]. The synthesis approach for the epoxysiloxane in our study is shown in Figure 1. We used the Rh catalyst instead of Pt-based catalyst because the latter can cause the ring-opening polymerization of epoxy groups and can lead to an undesirable gelled product [25]. PMHS (40 mmol of Si-H), VCHE (5.96 g, 48 mmol), toluene (30 ml), and chlorotris(triphenylphosphine)rhodium(I) (5.4 mg) were added to a 100-ml three-neck flask that was equipped with stirrer, thermometer, and reflux condenser. After chlorotris(triphenylphosphine)rhodium(I) was dissolved, the system was stirred at 35°C for 24 h. The temperature was then raised to 80°C, and the compound was constantly stirred until the end of the reaction (when the Si-H infrared absorption peak at about 2,150 cm^−1^ disappeared). The resulting liquid was purified *via* flash column chromatography using petroleum ether-ethyl acetate (10:1 volume ratio) as the eluent to yield a light yellow-colored compound. Infrared spectra and solution state hydrogen-1 nuclear magnetic resonance (^1^ H NMR) spectra were recorded *via* a Bruker Vector-22 (BRUKER AXS GMBH, Karlsruhe, Germany) Fourier transform infrared (FTIR) spectrometer and a Bruker DRX-500 spectrometer, respectively. In NMR characterization, all chemical shifts are reported in parts per million downfield from tetramethylsilane (TMS) using the residual protonated solvent as an internal standard (CDCl_3_, ^1^ H 7.29 ppm). The viscosity of the epoxysiloxanes and their mixtures was measured using HAAKE RheoStress 600 rotational rheometer from Thermo Fisher Scientific (Waltham, MA, USA). Young's modulus of cured epoxysiloxanes was examined by Series IX Automated Materials Testing System from Instron Corporation (Norwood, MA, USA). 

### Imprinting and pattern transfer

**Figure 2:**
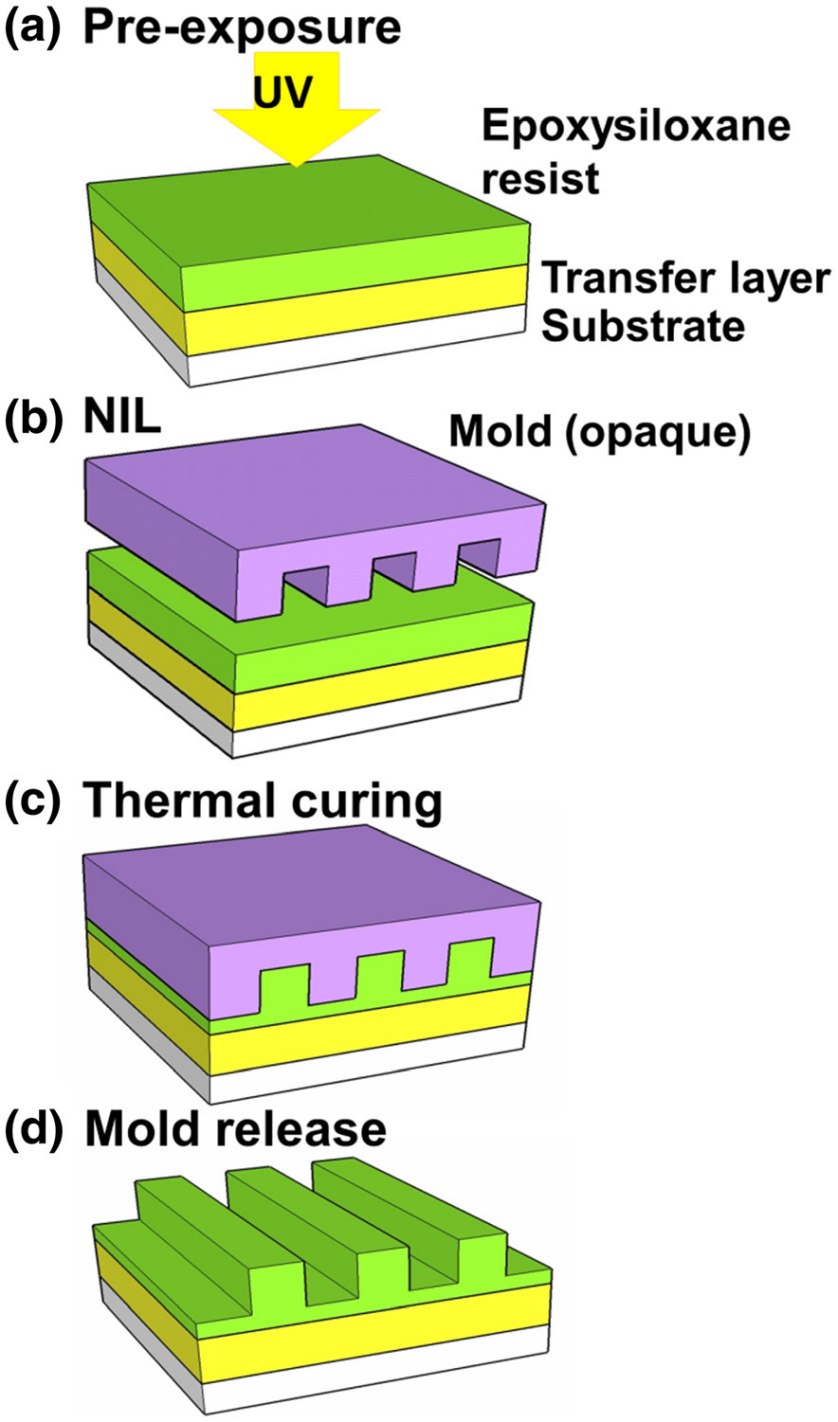
** Schematic illustration UV-assisted thermal curing imprint process.** (**a**) Preexposure of the epoxysiloxane resist by a cold UV-light; (**b**) imprint with a mold that can be opaque; (**c**) thermal curing the imprinted resist; and (**d**) mold release from the resist.

The synthesized epoxysiloxane was diluted in propylene glycolmethyl ether acetate (5 wt.%), and the solution was filtered before being used as a resist. The UV-assisted thermal curing NIL process is schematically illustrated in Figure 2. Uniform liquid resist films were spin-coated on bare silicon wafers with or without prebaked LOL as the transfer layers. The resist film was then exposed briefly to a UV light source (for example, the lamp used on a Karl Suss MA-6 mask aligner with 240 to 450-nm wavelengths and a cold light mirror) with an optimized UV dose of 300 mJ/cm [2] (Figure 2a). An imprint mold of quartz or silicon was then pressed into the thin resist film while the substrate was heated at 110°C for 30 s before mold separation. All imprint experiments were conducted in air. The etching of the epoxysiloxane resist and LOL was carried out in an Oxford RIE system. Typical etching recipes were 40 sccm O_2_, 3.0-mTorr pressure, 50-W power, −20°C (helium flow rate, 4.3 sccm) for LOL and 60 sccm CF_4_, 2.0-mTorr pressure, 40-W power, 20°C for the cross-linked resist films. 

### Cross-linking degree characterization

Solubility measurements of the resist films under various curing conditions were taken to check if the resist film was fully cross-linked. The measurement was simplified by rinsing the resist films with acetone after curing. Once fully polymerized, the thin film is insoluble in acetone due to the formation of highly cross-linked polymer network. An uncured or partially cured film would be stripped or destroyed by acetone [26].

## Results and discussion

**Figure 3:**
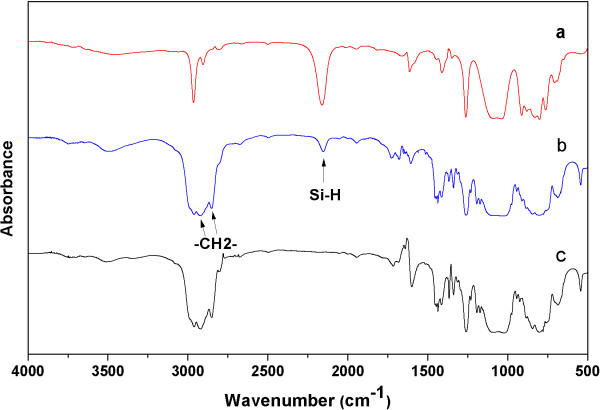
** FTIR spectra of PMHS polysiloxanes reacted with VCHE at different stages.** (**a**) Pure PMHS precursor; (**b**) reacted at 35°C for 24 h; (**c**) reacted at 35°C for 24 h and then at 80°C for 24 h.

**Figure 4:**
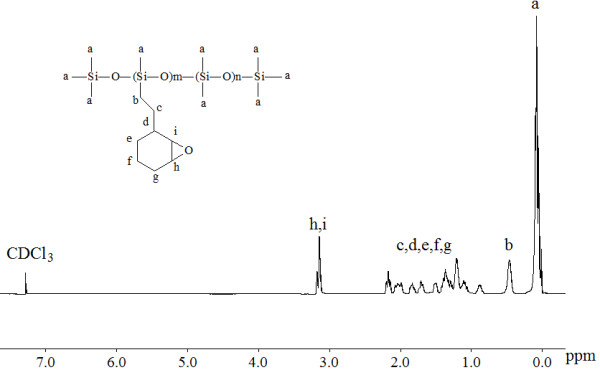
^** 1**^**H NMR spectrum of epoxysiloxane sample synthesized from PMHS with Si-H content of 50% (mole percentage).**

Figure 3 shows the FTIR spectra at different reaction stages for the hydrosilylation reaction of PMHS with VCHE. The adsorption peak at 2,150 cm^−1^ was attributed to the Si-H group. The absorption intensity decreased with the extension of reaction time, while new adsorption peaks appeared at 2,920 and 2,850 cm^−1^ attributed to the -CH_2_- group, suggesting that VCHE had been grafted onto the PMHS backbone by hydrosilylation. When the temperature was raised to 80°C and reacted for 24 h, the peak at 2,150 cm^−1^ disappeared, indicating the Si-H groups had been completely consumed by VCHE. Figure 4 represents the ^1^ H NMR spectrum of the epoxysiloxane oligomer synthesized from PMHS with a Si-H content of 50% (mole percentage). The characteristic groups on the chemical structure of the aimed product and their positions in the NMR spectrum were labeled. The spectrum illustrated a complete absence of Si-H proton peak at *δ* = 4.7 ppm, and the presence of the characteristic epoxide, Si-CH_2_- and Si-CH_3_ proton resonances at *δ* = 3.1 to 3.2, 0.47, and 0.08 ppm without peaks of methyl and methylene of oxyethyl group at *δ* = 1.28 and 3.65 to 3.71 ppm, respectively. It was confirmed that the aimed epoxysiloxane was successfully obtained, and the competing epoxide ring-opening reaction was avoided.

Using this synthesis approach, a series of multifunctional epoxysiloxane with varied viscosity and epoxy functionality were prepared. Various properties of different oligomers and their cross-linked films are listed in Table 1. Since the hydrosilylation proceeded to high conversion without the formation of by-product, desired epoxy functionality could be obtained from the starting PMHS with the same Si-H functionality. The viscosity and its growth range of the epoxysiloxane increased with the increase of Si-H content of the PMHS. This was attributed to the link of the epoxy groups to PMHS that increased not only the molecular weight, but also the polarity of the epoxysiloxane. The increase of epoxy content of the oligomers also strongly influenced the properties of their cured films. Higher cross-linking density hindered the movement of the chain segments and made the cross-linked matrix more rigid but brittle, which resulted in higher modulus and lower strain. The epoxide and its ring-opening product, ether, were polar groups, so the increase of epoxy content of the siloxane led to the increase of the polarity of its cured film and the decrease of the water contact angle. As shown in Table 1, the desired parameters of epoxysiloxanes and their cured film, such as viscosity, polarity, and mechanical strength, could be further optimized *via* mixing the epoxysiloxanes with different epoxy functionality and viscosity at different ratio.

**Table 1:** Material properties of synthesized epoxysiloxanes and their mixtures

**Epoxysiloxane**	**Si-H of PMHS (mol%)**	**Viscosity of PMHS (mPa s)**	**Viscosity of epoxysiloxane (mPa s)**	**Properties of cured films**		
				**Water contact angle (degrees)**	**Strain at peak (%)**	**Young's modulus (MPa)**
I	100	33	257	74.9	1.085	381.930
II	50 to 55	15	60	83.7	1.813	114.553
III	15	28	37	98.9	15.100	10.379
IV^a^	-	-	52	91.3	6.300	88.617
V^b^	-	-	112	80.6	1.232	225.063

^a^IV = 60 wt.% II + 40 wt.% III; ^b^V = 20 wt.% I + 80 wt.% II.

**Figure 5:**
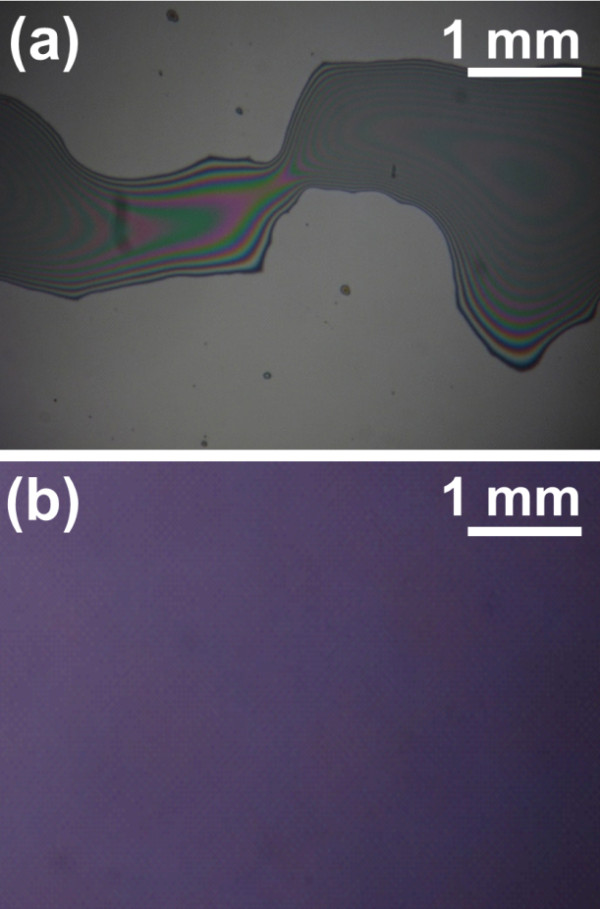
** Optical micrographs of epoxysiloxane resists on bare silicon wafer.** (**a**) Ruptured film of the epoxysiloxane resist with a viscosity of 37 mPa s and water contact angle of 98.9° formed in 10 min after spin coating and (**b**) uniform film of the epoxysiloxane resist with a viscosity of 112 mPa s and water contact angle of 80.6° kept unbroken for 1 h after spin coating.

**Figure 6:**
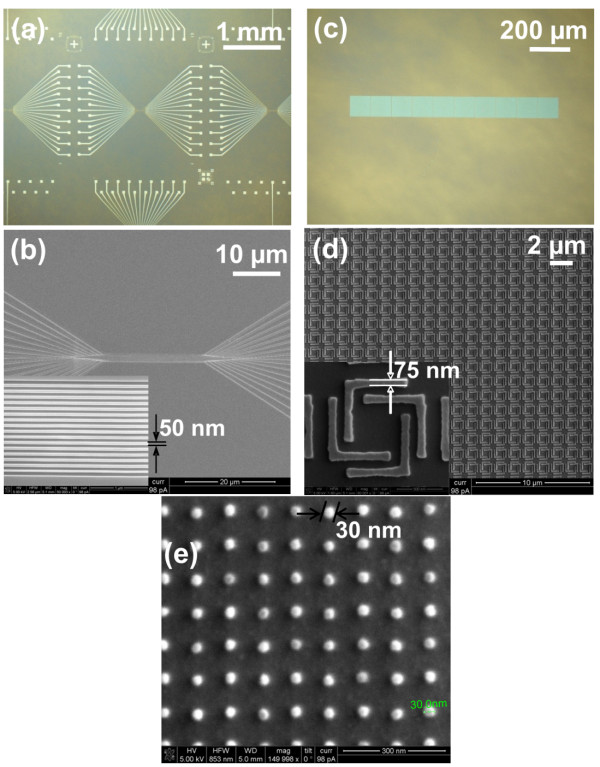
** Imprint results from the UV-assisted thermal curing NIL using the resist based on epoxysiloxane.** (**a**) Optical micrograph of the microscale features (50-μm pads and microscale fan-outs) that are connected to the nanowires. (**b**) SEM images of nanoscale fan-outs and nanowires. The inset shows 16 parallel nanowires with a feature size of 50 nm. (**c**) Optical micrograph of a 1-mm × 100-μm array of fourfold symmetric L-shaped features. (**d**) SEM image of part of the array. The inset is a zoom-in image of one fourfold symmetric L-shaped feature with the smallest feature size of about 75 nm and a height of 60 nm. (**e**) SEM image of 30-nm diameter dots in an array of 100-nm pitch.

Due to their insensitivity toward oxygen during curing, mechanical strength, and resistance to oxygen plasma after cross-linking, epoxy-containing silicone resins are highly attractive for making lithography resists [27,28]. We prepared NIL resists with the synthesized products, and the ingredients and their functions of a typical NIL resist formulation are listed in Table 2. In this resist, the multifunctional epoxy groups in the curable materials enable a high curing speed, a high cross-linking degree of the resist, and enhanced mechanical strength. Epoxysiloxane I bearing one epoxy group per repeating unit is a cross-linker to intensify the mechanical strength of the resist. Epoxysiloxane II with a moderate viscosity and epoxy functionality is used as a major ingredient to provide a proper mechanical strength and high oxygen RIE etching resistance. Epoxysiloxane III serves as a reactive plasticizer to reduce the brittleness of the cured resist film due to its high cross-linking density. In addition, the resist consists of a diaryliodonium photo-acid generator and an organic solvent as a diluent for spin coating. By adjusting the mixture ratio of different epoxysiloxanes, we fine-tuned the viscosity and polarity of the resist, which were critical to the film forming ability on a substrate. For example, it was difficult for a resist of too low viscosity and polarity such as Epoxysiloxane III to form a thin uniform film on a Si wafer by spin coating method (Figure 5a). Due to surface tension of the liquid film, the film ruptured into polygonal networks of liquid rims or sets of liquid droplets as have been observed in other resists [29-31]. However, a resist with optimized viscosity and polarity synthesized in this study formed a continuous and uniform film on the Si substrate after spin coating (Figure 5b). Nanoscale and microscale features with a variety of geometries have been faithfully imprinted into the resist films (Figure 6). First, groups of 50-nm half-pitch nanowires, 50-μm-wide contact pads, and the fan-out structure connecting them (1,000 times difference in lateral scale) were simultaneously imprinted into the resist using the same mold over a large area (Figure 6a, b). Second, complex nanostructures such as arrays of fourfold symmetric L-shaped features with sharp corners were imprinted with high uniformity (Figure 6c, d). The uniform color of the patterned region in the optical micrograph suggested minimal residual layer thickness variation over the 1 mm × 100 μm imprinted area (Figure 6c). The high fidelity duplication of the sharp corners (Figure 6d) without peel off proved that the resist has excellent cavity filling properties as well as easy release from the mold and strong adhesion to the substrate. Third, dot arrays of 100-nm pitch and 30-nm diameter were imprinted into the resist, demonstrating a 30-nm resolution. It is worth noting that the resolution was mainly limited by the feature size on the imprint mold, and it is possible to imprint features much smaller than 30 nm provided such a mold is available. 

**Table 2:** Ingredients of a typical NIL resist used in this study and their functions

**Ingredient**	**wt.%**	**Function**
I^a^	1	Silicon-containing oligomer; increases the viscosity and polarity of resist and provides high cross-linking density
II^a^	4	Silicon-containing oligomer; provides oxygen RIE etching resistance and moderate cross-linking density
III^a^	0.5	Silicon-containing oligomer; relieves mechanical properties as plasticizer
PGMEA	94.2	Low-viscosity diluents; improves resist flow for spin coating
Diphenyliodonium salt	0.3	Photo-initiator; generates cationic acids upon exposure to UV radiation

^a^I, II, and III are the same epoxysiloxane samples listed in Table 1.

**Figure 7:**
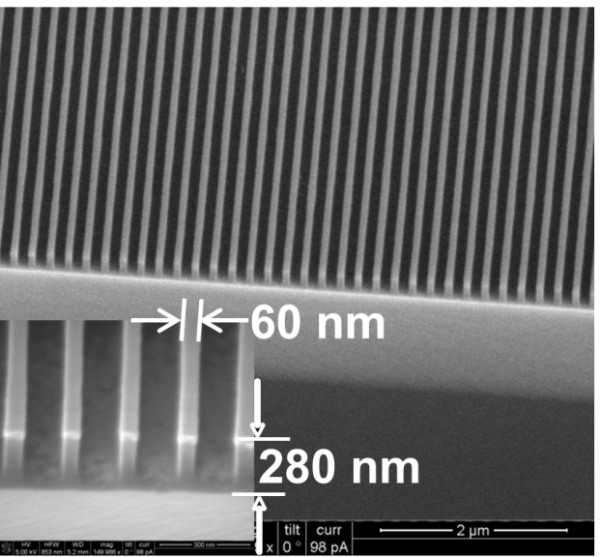
** High aspect ratio pattern transfer using two-step RIE.** The inset shows a high magnification SEM image of 60-nm-wide, 280-nm-high grating lines of 200-nm pitch in which the high aspect ratio (4.7) of the resist stacks is demonstrated. Samples were titled 45° for the cross-section imaging.

The cured resist exhibits excellent etching properties. The silicon-containing materials in the resist provide high resistance to O_2_ RIE, opening the opportunities for high aspect ratio nanostructure fabrication provided that a proper underlying transfer layer is used. We started with an 80-nm-thick resist film (see Table 2 for composition) on a 200-nm-thick LOL on Si, followed by UV-assisted thermal NIL using a mold of 200-nm pitch gratings (60-nm-wide and 82-nm-deep trenches). After removing the residual epoxysiloxane layer using RIE with CF_4_ gas, the LOL was etched using O_2_ plasma with the remaining epoxysiloxane as an etching mask. Figure 7 shows the 200-nm pitch etched epoxysiloxane/LOL lines of 60-nm width and 280-nm height (an aspect ratio of 4.7) that have vertical sidewalls (Figure 7).

**Figure 8:**
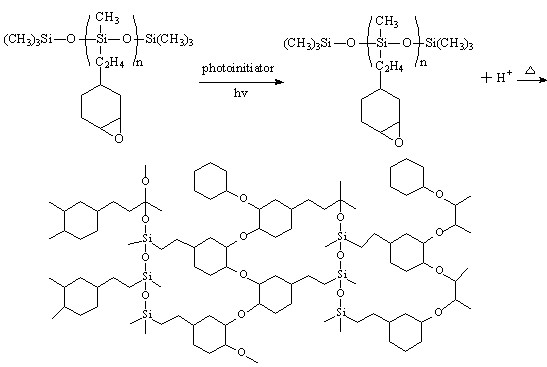
Formation of cross-linked polymer network by photo-initiated cationic polymerization of multifunctional epoxysiloxane.

Most currently available thermo-curable resists require lengthy curing cycles (e.g., epoxy or (meth)acrylate-based resists). This is because the thermally catalyzed polymerization for these materials is very slow. Our UV-assisted thermal curing process significantly shortens the curing time. In our current process, the UV exposure before thermal curing quickly generates a large amount of active cations in the resist through a mechanism as described in Figure 8. These cations facilitate the cross-linking of the resists through the ring-opening polymerization mechanism (Figure 8). Due to the long lifetime of the cations, the polymerization of the epoxy will continue effectively even when the UV light is removed. Furthermore, this process can be significantly accelerated by heating, leading to the full polymerization of the epoxy group in the resist within 30 s.

**Figure 9:**
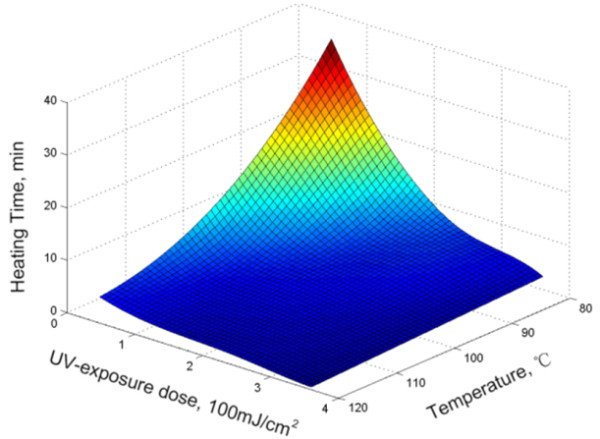
** The correlation of curing time with UV-exposure dose and heating temperature.** A higher UV dose for preexposure and a higher curing temperature greatly shorten the thermal curing time.

We have quantitatively studied the dependence of the thermal curing time on the temperature and preexposure UV dose for a 200-nm-thick resist, as shown in Figure 9. It is observed that a higher UV exposure dose and a higher temperature significantly reduce the time needed to fully cross-link the resist film. On one hand, without heating, the resist preexposed to UV radiation does not cross-link at all. This is because the conversion level of the cationic polymerization of epoxide was less than 10% as the reaction temperature below 50°C. Raising the polymerization temperature above 60°C caused a sharp increase in the conversion level [19,22]. On the other hand, without UV exposure, the resist only starts to polymerize at a temperature above 110°C, and the time needed to fully cross-link the resist is on the order of 10 min. The photo-acid generator, diaryliodonium salt, was thermally unstable at the temperature over 110°C and pyrolyzed into the same catalytic cation pieces to initiate the polymerization. While the pyrolysis of the photo-acid generators might be slower than the photolysis of it, thus, it took much longer time to fully cross-link the resist without UV radiation. Based on our experiments, we have determined the optimized dose to be the minimum dose (300 mJ/cm^2^) at which enough photo-acid generators are turned into cations. If the dose is too low, there would not be enough cations to initiate the polymerization of epoxy groups, while a higher dose is not necessary, and it actually leads to longer processing time for the imprint. 

Finally, in contrast to the photo-initiated radical polymerization, photo-initiated cationic polymerization can be implemented in the presence of oxygen. The resist also has long shelf time due to the fact that the propagating polymer cations are not reacting with themselves [12]. We have achieved similar imprint results with resists that were prepared a year earlier and have been kept in dark bottle at room temperature. This indicates that the shelf time of the resists is at least 1 year.

## Conclusions

We have synthesized a series of epoxysiloxane oligomers with controllable viscosity and polarity and developed a fast curable nanoimprint resist based on them. The new imprint resist was thermally cured within 30 s at a moderate temperature when a preexposure to UV light was adopted. Various patterns with feature sizes ranging from 30 nm to 50 μm were faithfully duplicated onto the resist film during this UV-assisted thermal curing NIL that was carried out in ambient air environment. The short cross-linking time was attributed to the large amount of cations that accelerated the thermal polymerization process. The unique properties of the resist had enabled a novel imprint process that combines the advantages of both thermal and UV-curable NIL such as low pressure, short processing time with a wider choice of imprint mold materials.

## Competing interests

The authors declare that they have no competing interests.

## Authors' contributions

JZ, XH, JZ, and YC carried out the synthesis and characterization of the epoxysiloxanes. QX carried out the nanoimprint and RIE experiments. HG, QX, WW, CY, and YC conceived the study and participated in its design and coordination. HG and QX drafted the manuscript. All authors read and approved the final manuscript.

## References

[B1] Chou SY Krauss PR Renstrom PJ Imprint of sub-25 nm vias and trenches in polymers Appl Phys Lett 1995 67 3114 10.1063/1.114851

[B2] Chou SY Krauss PR Renstrom PJ Imprint lithography with 25-nanometer resolution Science 1996 272 85 10.1126/science.272.5258.85

[B3] Chou SY Krauss PR Zhang W Guo L Zhuang L Sub-10 nm imprint lithography and applications J Vac Sci Technol B 1997 15 2897 10.1116/1.589752

[B4] Malaquin L Carcenac F Vieu C Mauzac M Using polydimethylsiloxane as a thermocurable resist for a soft imprint lithography process Microelectron Eng 2002 379 61 62

[B5] Liao W Hsu SL A novel liquid thermal polymerization resist for nanoimprint lithography with low shrinkage and high flowability Nanotechnology 2007 18 065303 10.1088/0957-4484/18/6/065303

[B6] Colburn M Johnson S Stewart M Damle S Bailey T Choi B Wedlake M Michaelson T Screenivasan SV Ekerdt J Willson CG Step and flash imprint lithography: a new approach to high-resolution patterning Proc SPIE Int Soc Opt Eng 1999 3676 379

[B7] Haisma J Verheijen M van den Heuvel K van den Berg J Mold assisted nanolithography: a process for reliable pattern replication J Vac Sci Technol B 1996 14 4124 10.1116/1.588604

[B8] Ge H Wu W Li ZY Jung GY Olynick DL Chen YF Liddle JA Wang SY Williams RS Cross-linked polymer replica of a nanoimprint mold at 30 nm half-pitch Nano Lett 2005 5 179 10.1021/nl048618k 15792435

[B9] Li Z Gu Y Wang L Ge H Wu W Xia Q Yuan C Chen Y Cui B Williams RS Hybrid nanoimprint-soft lithography with sub-15 nm resolution Nano Lett 2009 9 2306 10.1021/nl9004892 19422192

[B10] Schmid GM Miller M Brooks C Khusnatdinov N LaBrake D Resnick DJ Sreenivasan SV Gauzner G Lee K Kuo D Weller D Yang X Step and flash imprint lithography for manufacturing patterned media J Vac Sci Technol B 2009 27 573 10.1116/1.3081981

[B11] Austin MD Ge H Wu W Li M Yu Z Wasserman D Lyon SA Chou SY Fabrication of 5 nm linewidth and 14 nm pitch features by nanoimprint lithography Appl Phys Lett 2004 84 5299 10.1063/1.1766071

[B12] Decker C Photoinitiated crosslinking polymerisation Prog Polym Sci 1996 21 593 10.1016/0079-6700(95)00027-5

[B13] Jaworek T Bankowsky HH Koniger R Reich W Schrof W Schwalm R Radiation curable materials – principles and new perspective Macromol Symp 2000 159 197 10.1002/1521-3900(200010)159:1<197::AID-MASY197>3.0.CO;2-8

[B14] Kim EK Stacey NA Smith BJ Dickey MD Johnson SC Trinque BC Willson CGJ Vinyl ethers in ultraviolet curable formulations for step and flash imprint lithography Vac Sci Technol B 2004 22 131 135 10.1116/1.1635849

[B15] Cheng X Guo L Fu P Room temperature and low pressure nanoimprinting based on cationic photopolymerization of novel epoxysilicone monomers Adv Mater 2005 17 1419 10.1002/adma.200401192 34412429

[B16] Crivello JV Sasaki H Structure and reactivity relationships in the photoinitiated cationic polymerization of oxetane monomers J Macromol Sci, Pure Appl Chem 1993 A30 189

[B17] Olsson RT Bair HE Kuck V Hale A Photo-initiated cationic polymerization of a cyclo-aliphatic epoxy ACS Polym. Preprints 2002 42 797

[B18] Olsson RT Bair HE Kuck V Hale A Acceleration of the cationic polymerization of an epoxy with hexanediol J Therm Anal Calorim 2004 76 367

[B19] Pina-Hernandez C Guo LJ Fu P-F High-resolution functional epoxysilsesquioxane-based patterning layers for large-area nanoimprinting ACS Nano 2010 4 4776 10.1021/nn100478a 20731453

[B20] Wang P-I Bult J Ghoshal R Ghoshal R Lu T-M Rapid ultraviolet-curing of epoxy siloxane films Mat Chem Phys 2011 129 678 10.1016/j.matchemphys.2011.05.007

[B21] Ono Y Honda N Mori S Kono Y Sekiguchi A Experimental study of improved nano-imprint process by using SU-8 3000NIL resist J Photopolym Sci Technol 2006 19 393 10.2494/photopolymer.19.393

[B22] Youn SW Ueno A Takahashi M Maeda RP UV-assisted thermal imprint of SU-8 using nickel mold Int Conf Smart Manufacturing Application 2008 2008 389

[B23] Schuster C Reuther F Kolander A Gruetzner G mr-NIL 6000LT – Epoxy-based curing resist for combined thermal and UV nanoimprint lithography below 50°C Microelectron Eng 2009 86 722 10.1016/j.mee.2008.12.018

[B24] Boutevin B Youssef B Synthèse de polysiloxanes téléchéliques, 1. Synthèse de diols et diépoxydes Makromol Chem 1989 190 277 10.1002/macp.1989.021900206

[B25] Crivello JV Bi D The synthesis and cationic polymerization of multifunctional silicon-containing epoxy monomers and oligomers J Polym. Sci., Part A: Polym. Chem 1994 32 683 10.1002/pola.1994.080320407

[B26] Palmieri F Adams J Long B Heath W Tsiartas P Willson CG Design of reversible cross-linkers for step and flash imprint lithography imprint resists ACS Nano 2007 1 307 10.1021/nn7001079 19206681

[B27] Crivello JV Photographic method US patent no. 4081276 1978

[B28] Costner EA Lin MW Jen WL Willson CG Nanoimprint lithography materials development for semiconductor device fabrication Annu Rev Mater Res 2009 39 155 10.1146/annurev-matsci-082908-145336

[B29] Thiele U Velarde MG Neuffer K Film rupture by nucleation in the spinodal regime Phys Rev Lett 2001 87 016104 11461480 10.1103/PhysRevLett.87.016104

[B30] Xie R Karim A Douglas JF Han CC Weiss RA Spinodal dewetting of thin polymer films Phys Rev Lett 1998 81 1251 10.1103/PhysRevLett.81.1251

[B31] Vogler M Wiedenberg S Muhlberger M Bergmair I Glinsner T Schmidt H Kley EB Grutzner G Development of a novel, low-viscosity UV-curable polymer system for UV-nanoimprint lithography Microelectron Eng 2007 84 984 10.1016/j.mee.2007.01.184

